# The Propulsion Path of Synergy and Linkage Based on Artificial Intelligence and Digital Economy

**DOI:** 10.3389/fpsyg.2022.854542

**Published:** 2022-05-17

**Authors:** Yan Wu, Yuqin Zhu, Jingfeng Zhao

**Affiliations:** ^1^School of Economics and Management, Northwest University, Xi’an, China; ^2^School of Economics and Management, Hezhou University, Hezhou, China

**Keywords:** artificial intelligence, digital economy, collaborative linkage, advancement path, finance

## Abstract

From conception to birth, artificial intelligence inherited the power of human reproduction, such as creativity, self-improvement and language use, etc. These abilities cannot be exercised in other places. The purpose of this paper is to explore the path of collaborative promotion based on artificial intelligence and digital economy. First, it outlines the scattered use of data by individuals at the two application levels of the digital economy and the systematic use of data by enterprises on large organizations. In this study, artificial intelligence and digital economy are introduced into the ability of project information sharing in the field of the enterprise project, and then their important role in improving the performance of enterprise project management is analyzed. Secondly, try to use interviews, scale analysis and logical subtraction to formulate the measurement standards of the digital environment and project information publishing ability, and provide basic data for follow-up research. The experimental results show that 54% of middle-level personnel said that the synergy between enterprise artificial intelligence and digital economy is very good, and they have a positive attitude toward exploring new ways to promote digital economy integration in the field of artificial intelligence.

## Introduction

The Internet was first born in the United States as a military necessity, and then gradually became a political entity (Liu, 2018). After the third wave of upsurge, the Internet has become an infrastructure for reshaping all aspects of society and changing economic, social and cultural system (Niu, 2022). Nowadays, the Internet has gradually developed new technologies such as cloud computing, the Internet of Things, 5 g, etc., which is driving a new revolution (Drahokoupil and Jepsen, 2017). Not everyone is different from the internet, and economic model has become more common because of the internet. With the rapid development of global digital business, a new generation of information technology, represented by the Internet, big data and artificial intelligence is constantly emerging (Hassabis et al., 2017; Litvinenko, 2020). Becoming an important force to promote the development of characters and technological change. The digital economy and the real economy are constantly merging and develop, the era of the digital economy is coming. The digitization and in-depth development of the digital industry has led to the frequent integration of Internet users and the Internet industry. Big data collection has become a key factor of production and the main force of China’s economic development (Izbosarov, 2019; Pradhan et al., 2019).

With the rapid development of Internet information technology, a new round of technological revolution and industrial transformation is in full swing. Innovation and technological change are the main driving forces of economic development. At this critical moment of historical change and integration, artificial intelligence technologies that integrate data into systems, algorithms and computing power is growing all over the world (Akaev and Sadovnichii, 2019; Kopina and Kopin, 2019). New development space, new driving ability and future strategic directions will bring historic decline to the life and work of mankind. Digital marketing makes all information flow fully, solves the most complete information problems of original products, promotes supply chain orientation, improves system performance, maximizes system transformation and promotion, accelerates enterprise returns (Pei et al., 2019). At the same time, the development of the digital economy itself accelerates the rapid development of artificial intelligence, computer and other industries. Therefore, getting a higher position in the digital economy also means getting a higher position in the main links of the global economy and global enterprise chain (Zhang et al., 2021; Xu et al., 2022).

In recent years, artificial intelligence has developed rapidly at an alarming speed, and has gradually penetrated into human society, affecting people’s lifestyles, lifestyles and even promoting the transformation of economic structure, which has brought a huge impact on economic development. Related research has been paid more and more attention from all sectors of society. Simon C uses the financial databases to measure the market value of selected countries, and comparing them over time, so as to identify the digital economy companies. The results of the survey compare the market value of the digital economy, and the United States is in a leading position in both absolute and relative aspects. In terms of market value, the 11 largest companies are all American companies. For Germany, the results indicate that policy measures should be taken to improve competitiveness in this field. He provides a research method for measuring and comparing the digital economy between countries. This method can be applied to other countries that seek to benchmark their performance and derive policy measures in order to be able to compete with jurisdictions leading the digital economy (Simon et al., 2017). Alizadeh T discussed the launch of Google Fiber in Kansas City from three different perspectives, researched urban governance and Fiber projects-focusing on the numerous regulatory incentives and incentives provided for Fiber during the construction phase, discussed how the pre-existing digital divide and socio-economic inequality affected the Kansas City fiber plan. In order to better understand the geographical complexity of optical fiber services, a novel data mining technology and exploratory spatial data analysis were adopted to highlight the supply footprint of two counties in the metropolitan area of Kansas City (Alizadeh et al., 2017). It is of great practical significance to study the path of collaborative linkage promotion based on artificial intelligence and digital economy.

In this paper, the related science of the digital economy is used to study the promotion path based on the synergy of artificial intelligence and digital economy, and the influence of artificial intelligence on the Chinese enterprise system is explored. Research on spatial economy, geography and the new economic system shows that proximity is a key factor influencing external factors and a series of proximity effects. As a technology, artificial intelligence has the potential to spread technology and intellectual property to nearby regions. On this basis, the direct and indirect influence of artificial intelligence on local business systems is discussed from a global perspective.

## Propulsion Path of Synergy and Linkage Based on Artificial Intelligence and Digital Economy

### Digital Economy

Technological change is essentially a productivity revolution. If the core of the first and the second industrial revolution lies in the energy revolution (Makridakis, 2017). Then, the core of the third and fourth scientific and technological revolutions is to change the way, methods and efficiency of human information processing, thus leading to the improvement of all aspects of human society. This kind of innovation is data-centered, supplemented by high-speed networks and integration. And circuits and artificial intelligence have jointly created the prosperous digital economy in the world today. Specifically, the wide application of data is mainly reflected in two aspects:

(1) The scattered use of data by individuals

Before the industrial revolution, human beings seldom focused on recording their own data, and there was no condition to organize and structure this information. After the information technology revolution, with the emergence of home computers, human beings can record family bills, personal information and personal documents on personal computers. This kind of record is the most basic use of personal information (Chen et al., 2021). With the use of various sensors and the emergence of the mobile internet, various electronic terminals can collect various user information more acutely and quickly, and provide personalized services for users based on this information. For example, the watch can only provide health advice to users by monitoring the heart rate and the number of step of individuals. The use of this personal data reflects two characteristics:

First, the use of personal data by individuals is increasingly dependent on various enterprises businesses. This dependence is mainly due to two reasons. One is that individuals lack large-scale equipment for collecting and processing data, and the processing of personal data must rely on the enterprise’s servers and algorithms. The other is the deepening of the division of labor makes the satisfaction of individual needs depend on others. To obtain online shopping products, we must expose our geographical location and personal preferences to the e-commerce platform; In order to communicate with others instantaneously, we must expose our thoughts to instant messaging software (Zhao and Sun, 2020). This increasingly close connection has also damaged the privacy of users. Second, the use of personal data is mainly carried out by exporting itself. Because individuals use their own data mainly rely on various enterprises and organizations to process and analyze the data, and exchange data for corresponding services, individuals must export their own data to enterprises, governments and other organizations (Glauner et al., 2017).

(2) The systematic use of data by enterprises to large organizations

Before the industrial revolution, the government as the main and only large-scale organization in human society, had started large-scale data statistics. However, due to the limitations of technical level, organizational level and people’s cultural level, the data that the government can obtain mainly come from the national household registration, data, financial data, etc. These data types are relatively single, the scale is small, and they are prone to deviations in authenticity (Thrall et al., 2018). After the industrial revolution, the ability of enterprises to collect data has been strengthened day by day, and the quality and quantity of data have made a qualitative leap. However, these data can only be used as an auxiliary tool for the operation of human society. With the development of computer technology, human beings’ ability to collect and process large-scale data is constantly improving, and enterprises begin to use data on a large scale. From an overall point of view, the use of data by enterprises reflects the following characteristics:

First, the data used by the enterprise is mainly obtained by collecting external data, rather than its own data. Although companies also use their own data, more data is obtained from individual consumers and the external world with the help of various mobile terminals and sensors. Some consumers offer it to enterprises on their own initiative, while others obtain it by satellites, etc. Due to the wide range of sources and the large amount of data, it constitutes big data. Second, the enterprise’s use of data is no longer an auxiliary tool, but a core element. From the enterprise’s point of view, it is mainly to optimize production through data analysis, train artificial intelligence and algorithm invention through data, attract traffic to gain profits through data integration, processing and distribution, and so on. For example, Meituan integrates business data and then distributes business data to consumers to make profits. Third, the use of data by enterprises has the characteristics of high threshold and strong systematic. Because enterprises mainly deal with big data, the data scale is huge, and personal computers are not competent, so they must use high-performance supercomputers with various computer algorithms.

### Artificial Intelligence and Digital Economy

**FIGURE 1:**
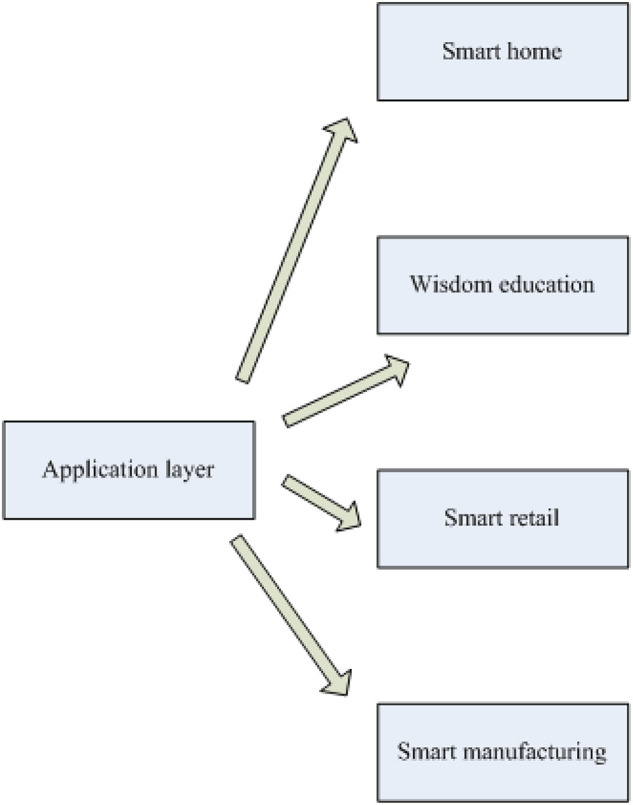
Framework diagram of artificial intelligence application layer.

The so-called digital economy is a series of economics with digital technology and information as the key production factors, modern information network as the key driving force, and efficient use of information and communication technology as an important driving force for productivity and economic planning. Data is an important commodity in the digital economy. The use of abundant data enables information services to surpass the speed of applications for the first time, reducing the information gap in economic services. The all-round digitalization of the economy is led by the dynamic growth of the digital economy. Understanding artificial intelligence promotes and promotes practices in the fields of medicine, education, transportation, retail, manufacturing and so on, which in turn affects the diversity of many fields in the social system, as shown in Figure 1.

First, the extensive use of data has promoted the exchange of supply and demand. In the traditional economy, supply chain and demand information must be generated by cost. Due to the limitation of time and space in the past trade, the efficiency of supply and the transmission of demand information were relatively low. Therefore, the Internet of Things allows all requests and quotations to be sent in time. One person can fully disclose quotations and requests on the e-commerce platform, and many sensors can also directly monitor demands and pain points. On this basis, the full exchange of supply and demand information gave birth to a new production mode based on the specific conditions and requirements of the system.

On the one hand, with the trade-offs based on digital platforms, data flow has evolved. Today, with the rapid flow of supply and demand information, anyone who has more supply and demand information can manage the economic foundation. All customers and suppliers will be sensitive to supply and demand information, which will be affected. Moreover, in the Internet of Things era, whoever can connect more information and data will have greater opportunities. Therefore, if a large amount of valuable information is connected, the company can become a site and continue to expand.

On the other hand, the digital platform has changed the status quo of large-scale integration of enterprise systems in the past. Due to the emergence of data and the continuous improvement of living standards, people have begun to pursue personalized needs. In this case, the economy began to rely on customer needs, and the path to meet diversified and hierarchical production needs by relying on the close cooperation between artificial intelligence and enterprises has become clear. The company has transformed from a B2C model to a C2B model, and implemented multi-channel distribution and flexible production based on low-cost data and fully integrated processes to quickly and timely meet market needs.

Second, the extensive use of data has effectively solved the problem of information asymmetry in the economy. Asymmetric information brings adverse selection and principal-agent problems in the economic field. Although information screening and some institutional methods have been put forward to solve these problems. However, these methods cannot really reduce the problems caused by information asymmetry. Through the extensive use of big data and artificial intelligence, these problems can be solved reasonably, for example, by comprehensively recording the past data of used cars. Reasonable evaluation of the price of second-hand cars, through video and audio monitoring and other means, can effectively solve the problem of subordinates’ failure.

Third, the combination of data and artificial intelligence technology has effectively improved the ability of data mining and analysis, increased the amount of information in all fields of the whole society and promoted the efficiency of the whole society. As far as the economic field is concerned, on the one hand, companies can collect more detailed information about consumers. For example, in the past, companies could only collect data on what consumers bought in our store, but now the e-commerce platforms can collect the browsing information of consumers. What product and what product take longer to build user portraits, analyze their customers’ preferences more deeply, and help to increasing sales. On the other hand, companies can also master their own production processes and supply chains data in more detail. Through the analysis of these data, they can find effective information to accelerate production and reducing inventory, thus improving economic efficiency. Correspondingly, the use of big data can also improve the government’s operational efficiency. At the same time, it can promote the acceleration of scientific research, the upgrading and progress of technology, thus improving the operational efficiency of the whole society.

Fourth, the widespread use of data promotes the growth potential of affiliated companies. On the one hand, due to the increasing frequency of data flow, storage and processing, it has driven the development of 5 g, WIFI, Bluetooth 5.0 and other technologies, also strongly promoted the development of the Internet communications industry. The demand for efficient storage has also driven the development of semiconductors, cloud storage and other industries, while the demand for data has also driven the potential development of algorithms such as artificial intelligence. On the other hand, high-efficiency information dissemination through data makes e-commerce, instant messaging, and word-of-mouth dissemination possible, thereby promoting the transformation and upgrading of the traditional economy, as well as the promotion of its business model.

**FIGURE 2:**
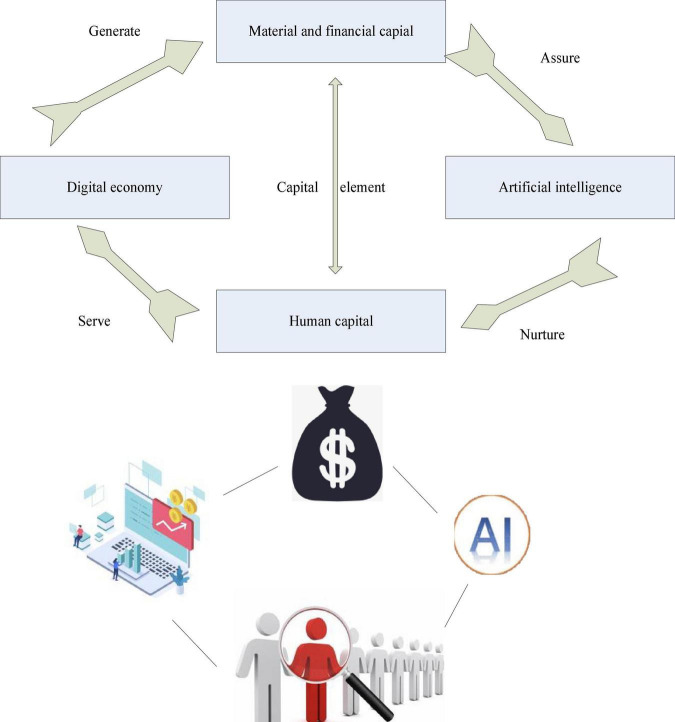
The “interactive symbiosis” logic of synergy between artificial intelligence and digital economy.

In short, an important feature of the digital economy is the large-scale expansion of data services. With the expansion of data services, information services increase and financial uncertainty decreases. This is the basic concept of digital programming. It can be seen that the application of data application itself is the driving force for the in-depth development of digital systems, it is also the data application for the in-depth development of the digital economy. Traditional intelligence and the digital economy take “capital” as the common point, linking self-reliance, self-confidence, and common sense of interaction, as shown in Figure 2.

### Construction of a Collaborative Development Model for Artificial Intelligence and Digital Economy

The existence of artificial intelligence makes knowledge grow at an explosive rate. Of course, artificial intelligence includes many technologies such as basic computing, speech recognition, visual recognition, big data and so on, but they undoubtedly play a great role in improving knowledge. The number of intelligent robots added to the index is used to describe the growth model of new knowledge. In the role of new knowledge, the exponential artificial intelligence capital Xt is added to describe the influence of artificial intelligence on knowledge and technology. Modifying the new knowledge growth mode:


(1) $$A_{t}=B\,{\left(a_{K}\,K_{t}\right)}^{\beta }\,{\left(a_{L}\,L_{t}\right)}^{\gamma }\,{\left(X_{t}\right)}^{\rho }\,A_{t}^{\theta },B>0,\beta \geq 0,\gamma \geq 0,\rho \geq 0$$

Finally, we assume that artificial intelligence plays a role of both substitution and productivity for labor, but under the continuous change of time, the effect of substitution and productivity affects the changing process.

Since it is assumed that the input of artificial intelligence capital plays two roles at the same time, Xt is used as the base number, but each effect has a different impact on output. Introducing two forces into the production function:


(2) $$Y_{t}=A_{t}\,{\left[\left(1-\alpha_{k}\right)\,K_{t}\right]}^{\alpha }\,{\left[\left(1-\alpha_{L}\right)\,L_{t}+X_{t}^{q}\right]}^{1-\alpha }+A_{t}\,X_{t}^{1-q}$$

Maximizing personal utility:


(3) $$c_{1,t}=\frac{1}{1+\beta }\,w_{t}$$

(4) $$s_{t}=\frac{\beta }{1+\beta }\,w_{t}$$

Corporate profits:


(5) ∏tAt[(1-aK)K]ta[(1-aL)Lt+Xtq]1-a+AtXt1-q-wtLt-RKt-tθKtt

Among them, Rt is the rate of return on traditional material capital. The first item on the right represents the company’s income, and the last three items are wages wtLt and the cost of traditional material capital RtKt. The maximization of corporate profits means that the following formula is established:


(6) $$\frac{\partial \,\prod_{t}}{\partial L_{t}}=A_{t}\,{\left[\left(1-a_{K}\right)\,K_{t}\right]}^{a}\,\left(1-a\right)\,{\left[\left(1-a_{L}\right)\,L_{t}+X_{t}^{q}\right]}^{-a}\left(1-a_{L}\right)-w_{t}=0$$

(7) $$w_{t}=\theta_{t}=\frac{\left(1-a\right)\,\left(1-a_{L}\right)\,A_{t}\,{\left[\left(1-a_{K}\right)\,K_{t}\right]}^{a}}{{\left[\left(1-a_{L}\right)\,L_{t}+X_{t}^{q}\right]}^{a}}$$

$$\frac{\partial \,\prod_{t}}{\partial K_{t}}=A_{t}\,{\left[\left(1-a_{L}\right)\,L_{t}+X_{t}^{q}\right]}^{1-a}\,a\,{\left[\left(1-a_{K}\right)\,K_{t}\right]}^{a-1}$$

(8) $$\left(1-a_{K}\right)-R_{t}=0$$

(9) $$R_{t}=\frac{A_{t}\,a\,\left(1-a_{K}\right)\,{\left[\left(1-a_{K}\right)\,K_{t}\right]}^{a-1}}{{\left[\left(1-a_{L}\right)\,L_{t}+X_{t}^{q}\right]}^{a-1}}$$

We have observed that the increase in traditional physical capital will increase the wage rate because it increases the productivity of workers. When the market is in equilibrium, there is no arbitrage relationship between artificial intelligence capital and traditional physical capital, which satisfies *R*_*t*_ = θ_*t*_. The equilibrium solution is:


(10) $$X_{t}^{q}=\frac{\left(1-a\right)\,\left(1-a_{L}\right)}{a}=K_{t}-\left(1-a_{L}\right)\,L_{t}$$

The stock of artificial intelligence capital satisfies the following formula:


(11) $$X_{t}^{q}=\max\{0,\frac{\left(1-a\right)\,\left(1-a_{L}\right)}{a}K_{t}-\left(1-a_{L}\right)L_{t}$$

The above formula will not be negative. If it is less than 0, the company will not invest in artificial intelligence capital. Bringing equilibrium conditions into the production function:


(12) $$Y_{t}=\frac{\left(1-a\right)\,\left(1-a_{L}\right)\,\left(1-a_{K}\right)}{a}\,K_{t}+\left[\frac{\left(1-a\right)\,\left(1-a_{L}\right)}{a}\right]K_{t}-\left(1-a_{L}\right)L_{t}]{}^{1/q}$$

In the OLG model, the capital stock in period t + 1 is equal to the savings in period t:


(13) $$S_{t}=S_{t}\,L_{t}\,K_{t+1}+K_{t+1}=L_{t}\,\frac{\beta }{1+\beta }\,\frac{\left(1-a\right)\,\left(1-a_{L}\right)\,A_{t}\,{\left[\left(1-a_{K}\right)\,K_{t}\right]}^{a}}{{\left[\left(1-a_{L}\right)\,L_{t}+X_{t}^{q}\right]}^{a}}$$

Dividing both sides by Lt + 1, and then bring in the equilibrium conditions and the production function, and we get:


(14) $$K_{t+1}+K_{t+1}=\frac{\beta \,\left(1-a\right)\,\left(1-a_{L}\right)\,A_{t}}{\left(1+n\right)\,\left(1+\beta \right)}\,{\left[\frac{a}{[\left(1-a\right)\left(1-a_{L}\right)}\right]}^{a}$$

In steady state:


(15) $$K_{t+1}=K_{t}=K$$

(16) $$k=\frac{\beta \,\left(1-a\right)\,\left(1-a_{L}\right)\,A_{t}}{\left(1+n\right)\,\left(1+\beta \right)}\,{\left[\frac{a}{[\left(1-a\right)\left(1-a_{L}\right)}\right]}^{a}-x_{t+1}$$

The equilibrium solution of per capita capital is not a constant, but is also affected by xt + 1, and xt + 1 is a function of K and L. Here, we only need to prove that K is not a constant. It is intuitive and the formula will not be expanded. The other interesting thing is that if:


(17) $$S_{t}=s_{t}\,L_{t}=K_{t+1}+X_{t+1}^{q}$$

So:


(18) $$\frac{\left(1-a\right)\,\left(1-a_{L}\right)}{a}\,k=\frac{\beta \,\left(1-a\right)\,\left(1-a_{L}\right)\,A_{t}}{\left(1+n\right)\,\left(1+\beta \right)}\,{\left[\frac{a}{\left(1-a\right)\,\left(1-a_{L}\right)}\right]}^{a}+\left(1-a_{L}\right)$$

Under the same conditions, K is still constant. It can be seen that if there is only artificial intelligence capital that can replace labor in the t + 1 period, the economy will stagnate. Once the productivity effect of artificial intelligence is introduced, the economy will not be able to reach a stable state. Per capita capital K will always be affected by the development level of artificial intelligence. New tasks will continue to be generated in the economy, and more new industries will absorb labor.

## Investigation and Research on the Propulsion Path of Synergy and Linkage Based on Artificial Intelligence and Digital Economy

### Company-Related Introduction

**FIGURE 3:**
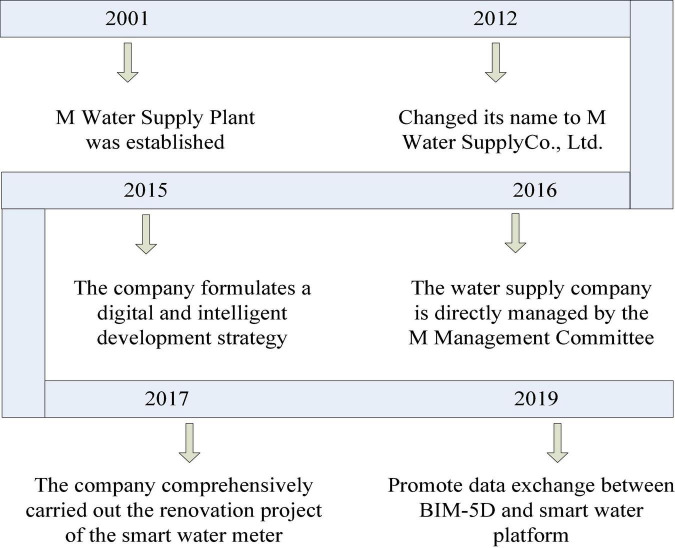
Memorabilia of M Water Supply Company.

M Water Supply Plant was established in 2001 and later changed its name to M Water Supply Co., Ltd. After 20 years of unremitting efforts, it has developed into a smart water company with smart Internet of Things artificial intelligence technology. As of the end of 2021, the water supply company has the ability to supply 200,000 cubic meters of water per day to the high-tech zone, and the total length of the water supply pipeline network is more than 200 kilometers. The major events of M Water Supply Co., Ltd., from 2001 to 2020 are shown in Figure 3.

### Case Study Interview Design

**FIGURE 4:**
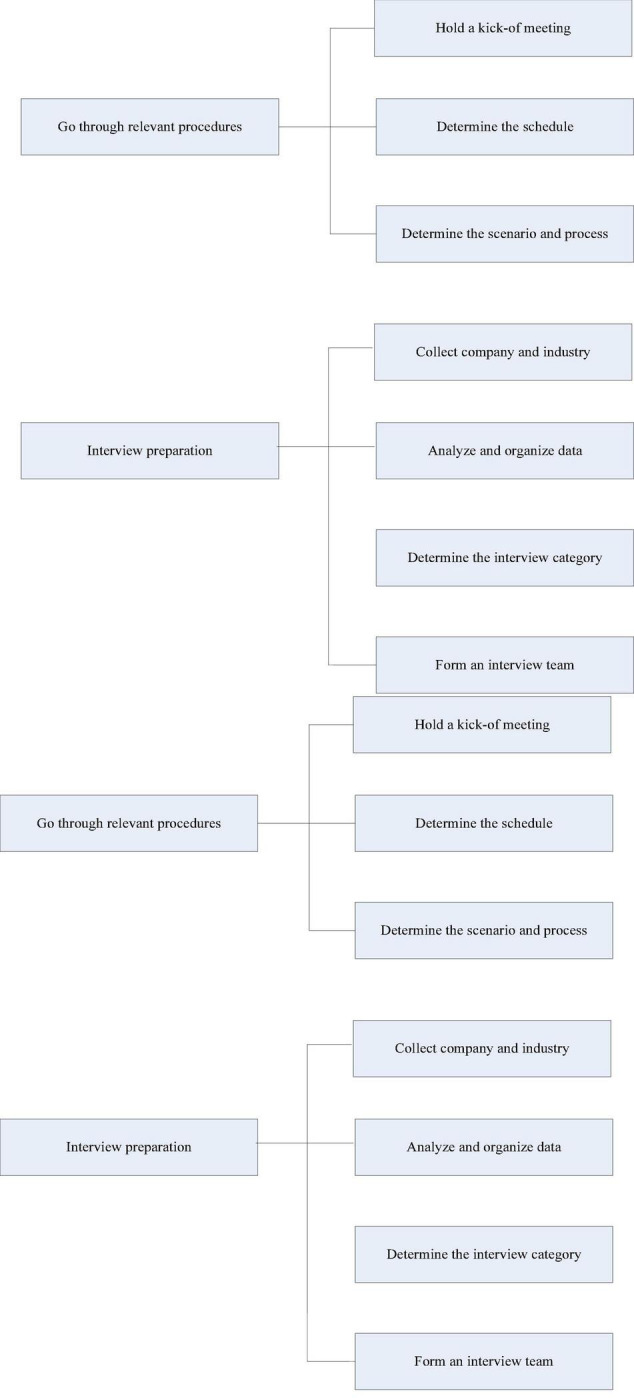
The main work of the preparation phase.

Convene a case study group discussion meeting to determine the interview item design, interview structure, and data collection and analysis related matters; coordinate with the company to determine the time schedule for each stage of the case interview investigation; Gaining an in-depth understanding of the basic situation of M Water Supply Co., Ltd., complete the formulation of the interview outline, initially complete the collection of primary and secondary data, and make preparations before the interview. The specific situation is shown in Figure 4. The questions investigated in this article include the evaluation of the synergy between enterprise artificial intelligence and digital economy, the overall management maturity of the company, and the evaluation of the efficiency of the company and its project management.

Before the start of the interview, first, let each respondent understand the purpose and basic requirements of this survey in advance, and the respondent needs to be prepared in advance. Secondly, open interviews and semi-structured interviews were conducted for each interviewee, and some questionnaires were filled in. Finally, arrange the interview sequence of different levels of personnel in the company. First, interviews employees, then interview middle-level leaders, and finally interviews the decision-making level. The total of 100 people were interviewed, A total of 50 people were interviewed, the specific situation is shown in Table 1.

**TABLE 1:** Interview and survey time schedule of M Water Supply Company.

Date	Time	Place	Task	Personnel
Monday	9:00–12:00	Mobilization meeting room	Plan management	Interview team and related persons in charge
	14:00–17:00	Interview meeting room	Semi-structured interview	Complete interviews with 3–4 people
Tuesday	9:00–12:00	Studio	Purification problem	All members of the interview group
	14:00–17:00	Interview meeting room	Semi-structured interview	Complete 5–7 interviews
Wednesday	9:00–12:00	Interview meeting room	Semi-structured interview	Complete 5–7 interviews
	14:00–17:00	Interview meeting room	Semi-structured interview	Complete 5–7 interviews

### Data Processing and Analysis

In this paper, SPSS 22.0 software is used to make statistical analysis on the results of the questionnaire, and the *T* test is carried out. The formula of *t* test used in this paper is as follows:


(19) $$t=\frac{\bar{X}-\mu }{\frac{\sigma \,X}{\sqrt{n}}}$$

(20) $$t=\frac{\bar{X_{1}}-\bar{X_{2}}}{\sqrt{\frac{\left(n_{1}-1\right)\,S_{1}^{2}+\left(n_{2}-1\right)\,S_{2}^{2}}{n_{1}+n_{2}-2}}\,\left(\frac{1}{n_{1}}+\frac{1}{n_{2}}\right)}$$

Among them, formula (1) is a single population test, x ^–^ is the sample average, s is the sample standard deviation, and n is the number of samples. Formula (2) is a two-population test, s_1^2 and s_2^2 are the variances of the two samples, and n_1 and n_2 are the sample sizes.

## Investigation and Analysis Based on the Collaborative Linkage Advancement Path of Artificial Intelligence and Digital Economy

### Coding and Analysis of Employee Interview Data

Through the investigation and interviews of a total of 33 employees of M Water Supply Company, after collecting and sorting out the data, 558 valid data were finally obtained. Through the coding methods shown in Examples 1 and 2, the 558 pieces of data of 33 employees were coded, 60 first-level coding concepts were obtained. Then, all the first-level coding concepts were coded, a total of 14 core concepts of secondary coding; Finally, all the concepts of secondary coding are further coded, and finally three levels of enterprise’s project information sharing ability, enterprise’s project management maturity and management efficiency of the enterprise and its projects are obtained. Selecting some data codes for display. Interview employee W of the company and coded the interview results, as shown in Table 2.

**TABLE 2:** Example of company employee interview data coding.

Data	Primary coding	Secondary coding	Three-level coding
The company’s prospects are good, regardless of the company’s and personal development are more confident	F 1–1 Project personnel’s evaluation of enterprise development	S 1–1 Satisfaction of the project implementation personnel of the enterprise	T 1–2 Management efficiency of the enterprise and its projects
I hope I can cooperate with the company’s strategy to provide better services	F 1–2 Work attitude of enterprise project personnel	S 1–2 Process control of enterprise projects	T 1–2 The maturity of enterprise project management
There is no special difficulty in work, can solve it by yourself or discuss and solve it with colleagues	F 1–3 Problem-solving ability of enterprise project personnel	S 1–3 Process control of enterprise projects	T 1–3 The maturity of enterprise project management

**FIGURE 5:**
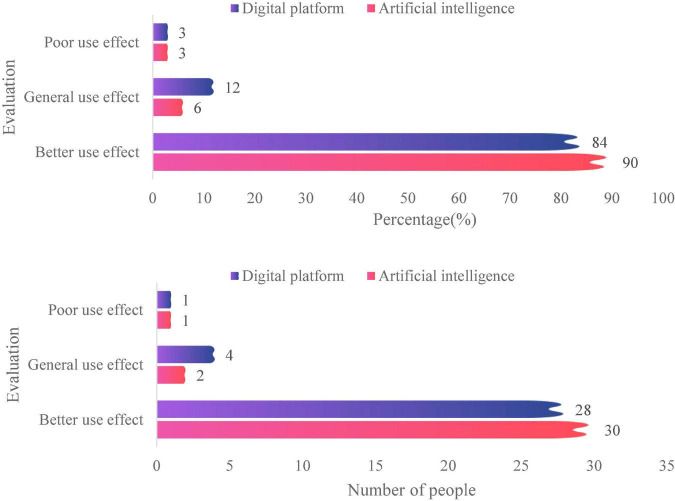
Evaluation statistics of enterprise employees on enterprise artificial intelligence and digital platforms.

After coding and sorting out all the interview data, perform summary statistics. By analyzing and processing the data, we get the results of employees’ evaluation of the company’s artificial intelligence. The data of all employees’ evaluations on the company’s digital platform are summarized and processed through data analysis and processing. Getting the evaluation results of enterprise employees on the enterprise digital platform, the specific content is shown in Figure 5. It can be seen that employees in enterprises have a high evaluation of the effect of using artificial intelligence, with 90% of employees receiving unanimous praise, and 84% of employees think that using the digital platform has a good effect.

**FIGURE 6:**
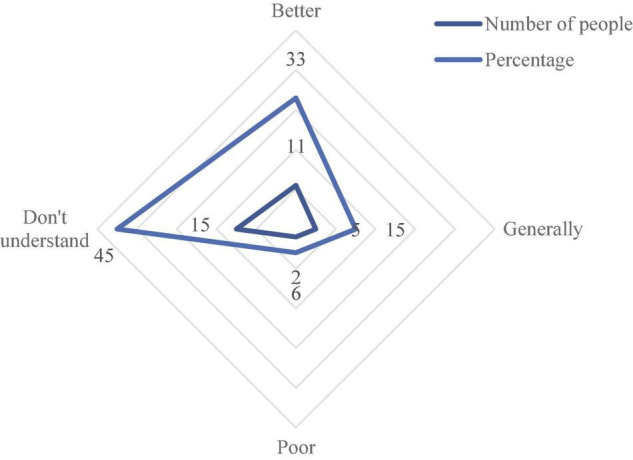
The evaluation statistics of corporate employees on the synergy of corporate artificial intelligence and digital economy.

The data of all employees on the synergy evaluation of enterprise artificial intelligence and digital economy is collected and statistics, and the evaluation result of synergy of enterprise artificial intelligence and digital economy is obtained through the analysis and processing of the data. The specific content is shown in Figure 6. A 11 people think that the synergy between enterprise artificial intelligence and the digital economy is very good, and 15 people don’t understand the effect.

### Coding and Analysis of Interview Data for Middle-Level Personnel

**FIGURE 7:**
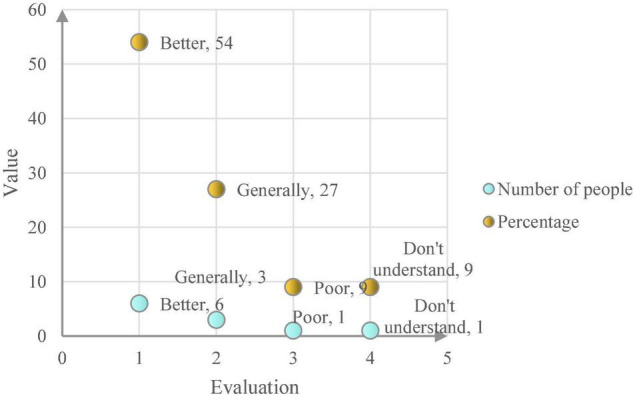
The evaluation statistics of mid-level employees on the synergy of enterprise artificial intelligence and digital economy.

After conducting surveys and interviews with a total of 11 middle-level managers of M Water Supply Company, after collecting and sorting out the data, a total of 200 valid data were finally obtained. Then, all the 200 pieces of raw data are coded in the form of coding examples, and all the data of the evaluation of the collaboration between enterprise artificial intelligence and the digital economy by middle-level personnel are collected. Through data analysis and processing, the information of middle-level personnel in enterprise artificial intelligence can be obtained. The evaluation results of the synergy with the digital economy are shown in Figure 7. Most middle-level people said that the synergy effect of enterprise artificial intelligence and digital economy is very good, while a few people thought that the effect is average, and two people said that the effect was poor and they don’t know the effect.

### Coding and Analysis of Interview Data for Senior Personnel

After conducting surveys and interviews with a total of 6 senior managers of M Water Supply Company, through collecting and sorting out the data, a total of 115 valid data were finally obtained. Then all 115 pieces of original data are coded in the form of coding examples. Finally, the data of all senior personnel on the synergy evaluation of enterprise artificial intelligence and digital economy is collected and statistics, and through the analysis and processing of the data, the evaluation results of the senior personnel on the synergy of enterprise artificial intelligence and digital economy are obtained. The specific content is shown in Table 3. The senior staff of the company think that the synergy between artificial intelligence and the digital economy is good, five of them think it is very good, and one thinks it is good.

**TABLE 3:** The evaluation statistics of corporate senior personnel on the synergy of corporate artificial intelligence and digital economy.

What is the effect of synergy between enterprise artificial intelligence and digital economy		
**Evaluation**	**Number of people**	**Percentage (%)**
Better	5	83
Generally	1	16
Poor	0	0
Don’t understand	0	0

### Summary Analysis of Case Interview Data and Verification of Research Conclusion

Through interviews with more than 50 employees and managers of M Water Supply Company, the interview data of all interviewers was finally summarized. The specific conditions are shown in Table 4. On average, 40, 47, and 32% of the companies think that the project management efficiency, the maturity of enterprise project management, and the ability of enterprise project information contribution of this intelligent IoT artificial intelligence technology enterprise are very good.

**TABLE 4:** Summary of interview data from M Water Supply Company.

Evaluation	Enterprise and project management efficiency	Enterprise project management maturity	Enterprise project information contribution ability
Better	40%	47%	32%
Generally	27%	16%	35%
Poor	16%	20%	31%

**FIGURE 8:**
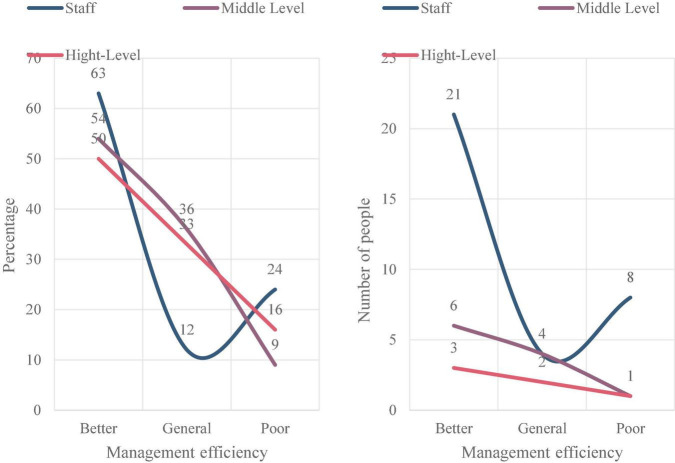
Enterprise and its project management efficiency.

**FIGURE 9:**
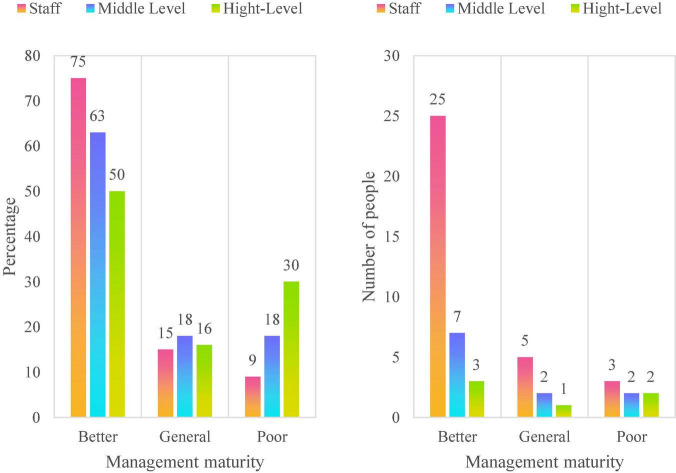
Enterprise and its project management maturity.

**FIGURE 10:**
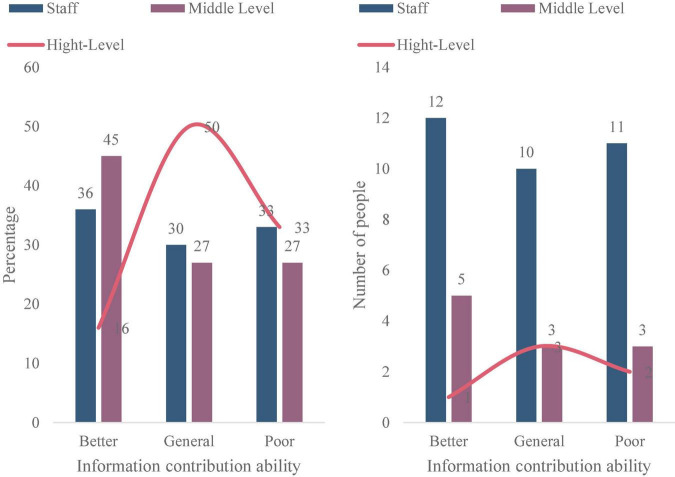
Enterprise project information contribution ability.

The corporate governance structure, management process and cultural construction of M Water Supply Company are relatively complete, and all aspects of management of the company are relatively mature. After decades of development, it belongs to an enterprise with high management maturity. After the summary analysis of the interview data, as shown in Figure 8, through the summary analysis of the company’s survey and interview data, more than 63% of the employees of M Water Supply Company have evaluated the efficiency of the company and its project management. It can be seen that in the years after the company was acquired, the efficiency of the company and its project management has been rapidly improved. The overall evaluation of the project management maturity of M Water Supply Company is higher. About 75% of the company’s employees evaluate the overall management maturity of the company, as shown in Figure 9. It can be seen that the maturity of enterprise project management has rapidly improved in the past few years. As shown in Figure 10, since the company started the strategic layout of smart water affairs, the ability of enterprise project information sharing has been greatly improved. About 45% of the middle-level evaluations of the project information contribution ability of M Water Supply Company is relatively good.

Before the acquisition of M Water Supply, the overall efficiency of the company, the success rate of the project, project and the satisfaction of all parties after the completion of the project began to decline seriously, and the level of management efficiency of the company was extremely low. However, since the company was acquired by HW Electronics, it has begun to build a smart water platform and start the second phase of the BIM management of the smart water plant. Through the analysis of the summary data, it can be found that after 4 years of development, the project management efficiency of M Water Supply Company has been significantly improved, but the maturity of enterprise project management has not changed much. This shows that companies are sharing project information. The development of capabilities has become the main influence path for the improvement of enterprise project management efficiency.

In general, the development course of M Water Supply Company can clearly reflect that the path of improving the efficiency of enterprise project management has changed obviously in the digital environment, and its performance in related practices is also higher than the related research results of this research. To a great extent, the degree of fitting has a good test and evaluation function on the empirical research conclusions, which verifies the related research conclusions.

## Conclusion

As the proportion of labor in many countries around the world has fallen sharply, artificial intelligence technology has solved the problems of underemployment or high labor costs in some automation industries, thereby maintaining and accelerating productivity growth at the macro and micro levels. First, the impact of artificial intelligence on the automation industry is obvious at both the macro and micro levels; at the micro level, companies are using different artificial intelligence technologies to give full play to their advantages, such as reducing labor costs, increasing productivity, improving quality, and reducing downtime. This article summarizes the following points based on the collaborative advancement path of artificial intelligence and digital economy:

First, artificial intelligence education system companies should respond responsibly to the challenges of rapid growth and transformation and progress in the digital industry, and fully meet the training requirements of the local digital industry based on the characteristics and development requirements of the local digital industry. Artificial intelligence talents, focus on the training of artificial intelligence talents has shifted from one position to a multidisciplinary environment, knowledge and skill opportunities have turned to undergraduate opportunities, traditional school sports training has turned to industry-school cooperation projects, fitness technology has turned to comprehensive quality, and the docking gradually meets the growth value of human capital in the digital industry.

Second, according to its own development status, closely integrate the development of the local digital industry, introduce the specific work of the digital industry, industry technology trends and industry performance requirements, and set up artificial intelligence talent training plans and curriculum settings.

Third, increase the research and development of cutting-edge technology projects. Finally, the artificial intelligence school should prepare high-quality artificial intelligence talent training reports every year, and integrate it with the overall development of building digital services. In order to meet the update and development of digital technology and information, digital information technology needs to revise professional and educational courses in time for the intelligent information provided by digital companies.

## Data Availability Statement

The original contributions presented in the study are included in the article/supplementary material, further inquiries can be directed to the corresponding author.

## Ethics Statement

Ethical review and approval was not required for the study on human participants in accordance with the Local Legislation and Institutional Requirements. Written informed consent from the patients/participants or patients/participants legal guardian/next of kin was not required to participate in this study in accordance with the National Legislation and the Institutional Requirements.

## Author Contributions

YZ: guiding the research directions and ideas. YW: writing and static analysis of data. JZ: experimental operation. All authors contributed to the article and approved the submitted version.

## Conflict of Interest

The authors declare that the research was conducted in the absence of any commercial or financial relationships that could be construed as a potential conflict of interest.

## Publisher’s Note

All claims expressed in this article are solely those of the authors and do not necessarily represent those of their affiliated organizations, or those of the publisher, the editors and the reviewers. Any product that may be evaluated in this article, or claim that may be made by its manufacturer, is not guaranteed or endorsed by the publisher.
